# Cell-free synthesis of amyloid fibrils with infectious properties and amenable to sub-milligram magic-angle spinning NMR analysis

**DOI:** 10.1038/s42003-022-04175-1

**Published:** 2022-11-09

**Authors:** Alons Lends, Asen Daskalov, Ansis Maleckis, Aline Delamare, Mélanie Berbon, Axelle Grélard, Estelle Morvan, Jayakrishna Shenoy, Antoine Dutour, James Tolchard, Abdelmajid Noubhani, Marie-France Giraud, Corinne Sanchez, Birgit Habenstein, Gilles Guichard, Guillaume Compain, Kristaps Jaudzems, Sven J. Saupe, Antoine Loquet

**Affiliations:** 1 Univ. Bordeaux, CNRS, Bordeaux INP, CBMN, UMR 5248, IECB, Pessac, France; 2 Univ. Bordeaux, CNRS, IBGC, UMR 5095, Bordeaux, France; 3 Zhejiang Academy of Agricultural Sciences, Hangzhou, 310021 China; 4 Latvian Institute of Organic Synthesis, Riga, Latvia; 5 Univ. Bordeaux, CNRS, INSERM, IECB, UAR, 3033 Pessac, France

**Keywords:** Protein aggregation, Supramolecular assembly

## Abstract

Structural investigations of amyloid fibrils often rely on heterologous bacterial overexpression of the protein of interest. Due to their inherent hydrophobicity and tendency to aggregate as inclusion bodies, many amyloid proteins are challenging to express in bacterial systems. Cell-free protein expression is a promising alternative to classical bacterial expression to produce hydrophobic proteins and introduce NMR-active isotopes that can improve and speed up the NMR analysis. Here we implement the cell-free synthesis of the functional amyloid prion HET-s(218-289). We present an interesting case where HET-s(218-289) directly assembles into infectious fibril in the cell-free expression mixture without the requirement of denaturation procedures and purification. By introducing tailored ^13^C and ^15^N isotopes or CF_3_ and ^13^CH_2_F labels at strategic amino-acid positions, we demonstrate that cell-free synthesized amyloid fibrils are readily amenable to high-resolution magic-angle spinning NMR at sub-milligram quantity.

## Abstract (web summary)

A method for cell-free synthesis of amyloid fibrils is reported that also allows for solid-state NMR analysis to characterize complex and pathological fibrillary assemblies.

## Introduction

Amyloids are structurally ordered protein aggregates that have attracted a lot of attention due to their crucial role in protein misfolding diseases^1^. These protein aggregates often show unbranched fibrillar morphology with a typical fibril diameter of several nanometers. Amyloid fibrils have been associated with several neurodegenerative diseases such as Alzheimer’s, Parkinson’s, Huntington’s, and prion diseases^2,3^. It is now clear that the amyloid fold is not exclusively restricted to misfolded proteins involved in pathological disorders, since the number of so-called functional amyloids is impressively growing^4^. Functional amyloids are protein aggregates often sharing the structural features of pathological amyloids but differing in their functions. Functional amyloids have beneficial roles in a cellular context, ranging from structural scaffolding in biofilm extracellular matrix^5^, formation of surface hydrophobins^6^, involvement in signaling processes^7^, or in innate immunity^8^. A paradigm to decipher the structure-function relationship of functional amyloids is the fungal HET-s prion^9,10^, for which a high-resolution structure was determined by solid-state NMR^11^.

Determining the three-dimensional (3D) structure of amyloid proteins in their fibrillar state has been a long quest and still poses important technical challenges^2,12^. Alongside with cryo-electron microscopy that has recently proven its capability to solve complex amyloid fibril structures^13–15^, solid-state nuclear magnetic resonance (NMR) is a powerful tool to investigate amyloid fibrils^16–19^, including for 3D atomic resolution structure determination^11,20–25^. Although solid-state NMR applied to protein samples mostly relies on the detection of ^13^C and ^15^N nuclei, the emergence of fast magic-angle spinning (MAS) and ^1^H-detected approaches^26–34^ have considerably expanded the toolkit of methods to decipher the structural organization of amyloid fibrils. These techniques take advantage of the excellent sensitivity of ^1^H spins—compared to ^13^C and ^15^N spins—and their high abundance in protein samples. Fast MAS probes are now available in many large-scale NMR infrastructures and multi-dimensional ^1^H-detected NMR fingerprints have been reported for deuterated^30,31,35^ and fully protonated^32,36–38^ protein samples. Recently, we have demonstrated that the recording of two-dimensional hCH experiments provides useful spectral fingerprints to characterize pathological^39^ and functional^25,40^ amyloids, in analogy to ^15^N–^1^H HSQC experiments traditionally used in solution NMR. Combined with a chemical shift analysis based on the Jardetzky database^41^, the use of such fingerprints is very valuable to rapidly evaluate the number of residues involved in the rigid amyloid core and secondary structure at amino-acid level resolution.

MAS NMR methods are limited by the poor sensitivity of ^13^C and ^15^N spins at natural abundance, requiring an enrichment to introduce NMR-active isotopes. This enrichment is usually achieved during the recombinant heterologous protein expression in *E. coli* by supplementing ^13^C- and ^15^N-labeled carbon and nitrogen sources. Amyloidogenic peptides and proteins can be difficult targets for expression in *E. coli*. Due to their hydrophobic character and high tendency to aggregate, the expressed amyloid proteins are often deposited as inclusion bodies^42,43^, requiring additional steps of chemical denaturing before the purification, and further refolding steps to isolate the active protein. Amyloid proteins can also present a high level of cytotoxicity when overexpressed, leading to poor production yields. In addition, the sample preparation often relies on a final step of in vitro protein aggregation, which can lead to additional structural polymorphism and thus broad NMR lines^44^. A powerful alternative to traditional *E. coli* expression is the cell-free (CF) expression technique. CF expression can overcome several limitations that exist during in vivo bacterial protein expression^45–47^. The CF expression system is safer than the production in bacteria, which requires strict control of the production environment and the technique allows for the incorporation of natural and unnatural amino acids^48–50^ that can be isotopically labeled at specific sites without severe metabolic scrambling^51^. Selective isotope labeling of CF-expressed globular and membrane proteins has already been successfully applied to solution NMR studies^52–55^. For solid-state NMR studies, CF expression has been used to investigate membrane proteins^56–61^. During the last decade, Böckmann and coworkers have optimized efficient CF production based on wheat germ extracts to explore the conformation of hepatitis B and C virus components by MAS NMR^62–66^. The main drawbacks of CF protein expression, in the context of MAS NMR studies, are the relatively low production yields and high cost of the large-scale preparation, which are typically required to apply ^13^C and ^15^N detection using 3.2 and 4 mm MAS NMR rotors (i.e., 10–50 mg protein samples are required). Because they require only sub-milligram amounts of samples, ^1^H detection methods at fast MAS regime in small diameter rotors circumvent the low yield problem^67^. Concurrently, ^19^F MAS NMR detection of fluorinated probes in CF-synthesized proteins may represent a complementary sensitive NMR approach to study protein aggregation and amyloid fibril conformation, provided that the introduction of fluorinated amino acid residues retains the canonical structure of the amyloid protein.

In this work, we present the production of CF-synthesized amyloid fibrils for MAS NMR. Our approach takes advantage of CF synthesis to incorporate NMR-active isotopes/labels at strategic positions. We demonstrate that the HET-s (218–289) prion can be readily assembled in the biologically relevant infectious amyloid form in the CF reaction mixture, recovered to high purity without the requirement of denaturing steps and purification, leading to a considerable simplification of the sample preparation process. Our approach is illustrated with the production of the CF-synthesized HET-s (218–289) amyloids with tailored ^13^C and ^19^F nuclei labeling amenable to high-resolution MAS NMR investigation using sub-milligram sample quantity.

## Results and discussion

### Production of cell-free synthesized HET-s

**Fig. 1:**
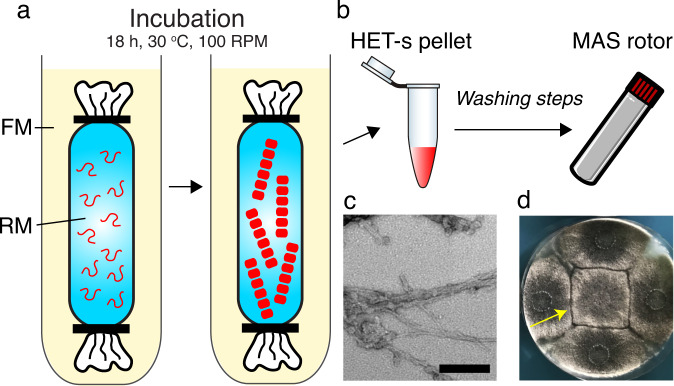
Cell-free synthesis of amyloid proteins, illustrated with the prion-forming domain of HET-s. **a** Schematic representation of the production of cell-free synthesized HET-s amyloid fibrils. RM: reaction mixture. FM: Feeding mixture. **b** RM is harvested using centrifugation and washed to obtain the NMR sample. **c** Electron micrograph of CF synthesized HET-s PFD amyloid fibrils. Scale bar: 100 nm. **d** CF-synthesized HET-s PFD triggers the phenotypic conversion of [Het-s*] strains (infection) detected as a regulated cell death reaction as observed by the barrage reaction (yellow arrow) when the focal strain in the center of the plate is confronted to the four [Het-S] strains at the periphery.

To develop a CF synthesis approach for amyloid proteins, we selected the HET-s protein. HET-s (218-289) is a prion-forming domain (PFD) from the filamentous fungus *Podospora anserina*, and it displays amyloid properties^9^. The HET-s PFD behaves as a prion in vivo, meaning that it can adopt a self-replicating infectious amyloid fold which is transmitted from cell to cell by cytoplasmic contact^10^. ^15^N/^13^C^11,68^ and ^1^H^69,70^ solid-state NMR chemical shifts are known. Purified HET-s PFD can self-assemble in vitro into infectious and non-infectious polymorphic fibrillar forms^11,71^ depending on aggregation conditions. The HET-s (218–289) gene fragment was incorporated into pIVEX vector 2.3 under the T7 promoter^72^. CF synthesis is a protein synthesis method performed in vitro without the requirement of living cells. In turn, transcription and translation processes are achieved in a mixture composed of cell extracts supplemented with amino acids, DNA, nucleotides, an energy regenerating system, salts and co-factors to achieve in vitro expression without the cell membrane physical barrier^73^. The CF protein synthesis was conducted using a dialysis method^74^ at 30 °C for 18 h with an *E. coli* S30 extract (schematically shown in Fig. 1a). The CF reaction mixture (RM) was dialyzed against the feeding mixture (FM) based on the components shown in Supplementary Tables S1–S3. After the CF reaction, the RM was harvested from a dialysis bag by centrifugation, and the pellet was then washed 3 times with H_2_O (schematically shown in Fig. 1b). This straightforward procedure allowed us to isolate the aggregated protein sample from magnesium pyrophosphate salts and nucleic acids. We observed that CF-synthesized HET-s PFD was pure, as assessed by SDS-PAGE (Supplementary Fig. S1). Notably, no specific columns or other methods besides washing with H_2_O were used during the purification procedure.

### Spontaneous assembly of HET-s aggregates in the CF mixture

HET-s monomers self-assembled directly in the RM, and we observed the formation of a white pellet. We hypothesized that CF-produced self-assembled HET-s(218-289) in the RM might resemble structurally HET-s amyloids obtained with standardized protocols using heterologous expression systems like *E. coli*., and might display mechanistic features of protein aggregation usually observed during the formation of deposits in bacterial inclusion bodies^75,76^. The formation of inclusion bodies is still a largely unknown process^77^, but it has been reported that they can have amyloid-like properties^78^. In the case of HET-s (218–289) it was even shown that bacterial inclusion bodies directly contain the biologically relevant amyloid structure^79^. In the context of CF-production of HET-s, this spontaneous aggregation might thus prove advantageous in that the pellet of HET-s aggregates can be directly retrieved from the RM (Fig. 1b). Indeed, the classical protocol of in vitro HET-s production and assembly^80^ includes a denaturing step after purification to unfold all HET-s monomers, prior to the concentration and aggregation steps. We therefore analyzed the properties of these spontaneously assembled aggregates, formed during the CF expression procedure.

It has been demonstrated that amyloid proteins are prone to undergo structural polymorphism during their aggregation^81,82^, at the level of their residue conformation (ultra-structural polymorphism) and/or at the level of the quaternary protofilament arrangement (segmental and packing polymorphism). Depending on the assembly conditions, HET-s PFD can also exist as distinct fibril structural polymorphs^11,71,83^ that are associated with infectious or non-infectious properties. Therefore, we asked the question, concerning the spontaneous protein assembly in the RM, are the CF-synthesized HET-s aggregates similar to those obtained in vitro under well-controlled buffer conditions? First, we performed electron microscopy on the HET-s PFD pellet and observed aggregates with fibrillar morphology, comparable to in vitro fibrils of HET-s obtained at pH 7 (Fig. 1c) using standard procedures^80^. Thus, the spontaneous formation of CF-synthesized HET-s in the RM leads to fibrillar assemblies with a well-defined morphology. Next, we investigated if CF synthesized HET-s aggregates are associated with HET-s prion infectivity. We conducted a prion infectivity assay using a fraction of CF-produced HET-s aggregates. We found that CF-synthesized HET-s aggregates successfully induced the formation of the [Het-s] prion in vivo in *P. anserina*^80^ (Fig. 1d) and thus possess the same infectious behavior as fibrils assembled in vitro from purified HET-s PFD^84^. Interestingly, the same observation was reported for HET-s PFD aggregates extracted from bacterial inclusion bodies and aggregates formed during heterologous overexpression in yeast^79^. Overall, our results indicate that CF-synthesized HET-s (218–289) aggregates display similar morphology and infectivity as HET-s PFD fibrils assembled from heterologously purified proteins.

The HET-s PFD pellet was collected after a period of 18 h, and protein expression was already visible after a period of 6 h. We explain such a fast protein translation process by the fact that *E. coli* CF protein synthesis has been previously reported to have an accelerated translation rate compared to eukaryotic expression systems^45^. The yield of CF HET-s synthesis was ~1 mg/mL, which is comparable to CF synthesis of integral membrane proteins from S30 *E. Coli* extracts reported by Dötsch and coworkers^85^. This pellet quantity is amenable to the filling of small-size solid-state NMR rotors such as 0.7 and 1.3 mm without too much loss of material.

### CF-synthesized amyloid fibrils are folded into the canonical β-solenoid structure

**Fig. 2:**
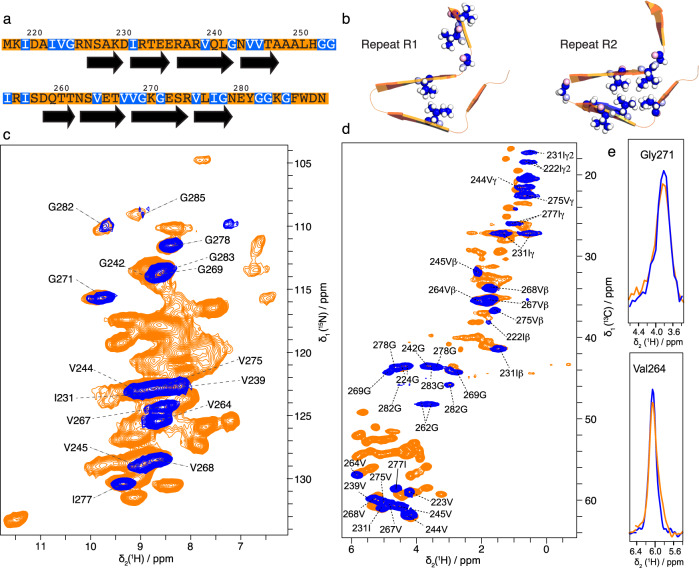
Magic-angle spinning solid-state NMR analysis of cell-free synthesized amyloid fibrils. **a** Primary sequence of HET-s (in orange), NMR-active isotope (^13^C, ^15^N)-labeled in the CF-GVI-HET-s sample are colored in blue. **b** Structure of the repeats R1 and R2 of HET-s PFD amyloid fibrils, G, V, and I residues are shown as spheres with carbon atoms colored in blue. **c** 2D hNH spectra of R-HET-s (orange) and CF-GVI-HET-s (blue) recorded at 100 kHz MAS, **d** 2D hCH experiments on R-HET-s (orange) and CF-GVI-HET-s (blue) recorded at 100 kHz MAS. **e** 1D ^1^H trace extracted from 2D hCH spectrum for residue G271 and V264.

To investigate whether the CF synthesis could be applied to study amyloid fibrils by MAS NMR or not, we designed a strategic labeling approach. The amyloid core of HET-s (218–289) represents a β-solenoid fold composed of two layers of β-strands, called the repeats R1 (N226-G242) and R2 (N262-G278). R1 and R2 are separated by an unstructured and mobile loop that is not detected in cross-polarization-based MAS experiments^86^. We selected three amino acids—namely glycine, valine, and isoleucine—to implement the labeling of NMR-active isotopes in the CF synthesis protocol for several reasons: (i) the HET-s PFD amyloid core is primarily made of hydrophobic contacts between valine, isoleucine and alanine residues^11,68^, (ii) selecting these three amino acids allows to cover most parts of the sequence, including R1, R2, and the unstructured regions (Fig. 2a, b) and (iii) ^13^C chemical shifts for valine, glycine, and isoleucine are distinguishable. We carried out the CF synthesis of a HET-s PFD sample (called here CF-GVI-HET-s) by incorporating (^13^C,^15^N)-labeled Gly, Val and Ile using 1 mL of RM. To obtain a favorable filling efficiency from the yield of CF production, we employed a 0.7 mm MAS rotor that was filled with ~500 μg of material. The 2D hNH experiment recorded at 100 kHz MAS (Fig. 2c, in blue) is composed of well-resolved correlations and we identified seven glycine, seven valine, and two isoleucine correlations. To examine the local conformation of CF-GVI-HET-s, we compared to a fully (^13^C,^15^N)-labeled HET-s sample (Fig. 2c, in orange) produced from recombinant expression and purification and in vitro aggregation (sample called R-HET-s). This sample is used to obtain the reference infectious form of HET-s PFD amyloid fibrils^71,84^. We observed that the chemical shifts for the G, V, and I residues in CF-GVI-HET-s coincide well with those obtained on R-HET-s. This indicates that the local residue conformation of HET-s aggregates obtained after the CF synthesis is comparable to the conformation of the reference infectious form of HET-s amyloid fibrils. Moreover, we observed a unique set of resonances in CF-GVI-HET-s, indicative of a very low structural polymorphism at the residue level. The same observation was made for the R-HET-s sample. Together, these results suggest that the folding and assembly process of HET-s monomers that is taking place in the cell-free RM leads to fibrillar aggregates that adopt the same unique conformation as observed in the infectious amyloid form of recombinant HET-s. This agreement is quite remarkable, considering that the RM is highly crowded and not purified, compared to in vitro conditions where HET-s monomers are very pure in a simple buffer with a controlled pH. The observation of a unique set of resonances also suggests the absence of metabolic scrambling for Gly, Val, and Ile residues. Isotope scrambling is usually less severe in CF synthesis^52,87^, although ^15^N isotope scrambling for Asp, Asn, Gln, and Glu have been previously reported for *E. Coli* CF systems^88,89^. Our results indicate that the CF synthesis protocol applied to amyloid proteins can efficiently reduce undesired metabolic pathways that might have led to the observation of additional correlations not belonging to G, V, and I.

### CF-synthesized amyloid fibrils allow strategic ^13^C isotope labeling to access NMR structural fingerprints with ^1^H detection

Next, we investigated the advantages of combining MAS ^1^H detection and the strategic incorporation of NMR-active isotopes to CF-synthesized amyloid fibrils. 2D hNH (Fig. 2c) and hCH (Fig. 2d) experiments on CF-GVI-HET-s were recorded in 1.5 h and ~6 h, respectively, on an 800 MHz spectrometer. These results show the recording of sensitive and well-resolved 2D experiments of CF-synthesized amyloid fibrils using sub-milligram quantities. We used two polarization transfers (^1^H to N/C followed by N/C to ^1^H) based on CP steps to filter residues belonging to the rigid core of the HET-s fibrils. Consequently, 4 residues (I219, I222, V223, and G224) of the unstructured N-terminal domain are filtered out, as well as residues G252, G253, I254, and I256 located in the unstructured segment between R1 and R2. All rigid residues expected from the amyloid core of HET-s PFD are observed in the 2D hNH spectrum (Fig. 2c). In addition, G282 and G283 are also observed, although they are not part of the rigid core. The ^15^N chemical shift dispersion is sufficient to distinguish all correlations, except for the pairs G283/G269, V244/I231, and V239/V275.

The 2D hCH experiment (Fig. 2d) allows for a better spectral dispersion due to the higher dispersion of the ^13^C dimension. The NMR experiment was designed to probe all kinds of ^13^C signals, including backbone and side chain resonances. The three pairs above could be distinguished based on their distinct Cα chemical shifts. Because our isotope-labeling strategy incorporates fully ^13^C labeled amino acids, the 2D hCH experiment gives access to side chain one-bond HC correlations for valine and isoleucine (no side chain for glycine). This information is useful to (i) distinguish residues that might have very close Cα chemical shifts and (ii) give access to Cβ chemical shifts, which can be used to compute the so-called secondary chemical shift values together with Cα^90^. As observed for ^15^N resonances, ^13^C chemical shifts are unique, indicating a very low level of structural polymorphism in the CF preparation.

An overlay of 1D ^1^H projections for selected residues is shown in Fig. 2e. We measured amide ^1^H line widths from 2D hNH spectrum (shown in Fig. 2c) for each resolved residue. Overall, ^1^H line-widths for CF-GVI-HET-s are ~268 Hz (±78 Hz), and for R-HET-s ~394 Hz (±87 Hz) (Supplementary Table S4). The homogenous line-width calculated from ^1^H T’_2_ coherence lifetime is ~213 Hz for CF-GVI-HET-s and ~250 Hz R-HET-s. These values indicate a comparable structural homogeneity for both samples. These line widths compare even more favorably for CF-GVI-HET-s since the CF RM was not pure, contrary to R-HET-s. A similar trend was observed for ^13^C-bonded ^1^Hα as measured in the 2D hCH spectrum, with ~225 Hz (±90 Hz) for CF-GVI-HET-s and ~223 Hz (±29 Hz) (Supplementary Table S5) for R-HET-s. This observation demonstrates the high quality of the CF-synthesized amyloid fibrils and suggests its applicability to more sophisticated ^1^H-detected MAS NMR experiments, since ^1^H nuclei are more sensitive to structural heterogeneity compared to ^13^C nuclei^62^.

To investigate the use of CF-synthesized amyloid fibrils to obtain MAS NMR experiments of higher dimensionality for ^13^C and ^15^N nuclei, we employed 3D spectroscopy to acquire hCCH TOCSY and hNCaH experiments. We used a 3D hCCH TOCSY to establish intra-residue ^13^C–^13^C–^1^H correlations (Supplementary Fig. S2a), this sequence being efficient to encode ^13^C resonances to their directly bonded ^13^C–^1^H pairs^32^. The choice of the labeling strategy is advantageous since Gly, Val and Ile have distinguishable ^13^C–^13^C side chain fingerprints. To complement the information available in the 3D CCH experiment, we tested the use of a 3D hNCaH. Because the CF-labeling strategy is based on the incorporation of labeled amino acids, one limitation in 2D hCH and hNH experiments could be the close proximity of CH and NH chemical shift pairs belonging to the same residue type (here G, V, and I). The 3D hNCaH experiment facilitates a better spectral decongestion, as exemplified with the residue V244 (Supplementary Fig. S2b), a residue not well distinguished in 2D hCH and hNH spectra but that can be spectrally isolated using a combination of 3D NCH and hCCH experiments with GVI isotope labeling. Overall, these 3D spectra exhibit less spectral overlap, simplifying the chemical shift assignment procedure. The model system used in our study is a medium size protein for solid-state NMR investigation, and the use of such 3D experiments will be even more beneficial for larger systems.

### ^19^F detection of CF synthesized amyloid fibrils

^19^F nuclei have numerous spectroscopic advantages for NMR^91,92^; they are NMR-active, sensitive, and 100% naturally abundant. ^19^F chemical shifts are very sensitive to the local environment and offer a useful tool for biomolecular NMR studies^91,93,94^. Moreover, because ^19^F nuclei are not naturally present in proteins, they provide local probes that can be NMR-detected without perturbing the spectral signal background. Structural investigation of proteins using ^19^F MAS NMR were mostly conducted with fluorines incorporated at the side chains of aromatic amino acids^95^. The fluorine-substituted aromatic rings additionally induce a strong contribution to the ^19^F chemical shift anisotropy (CSA) tensor, leading to an overall line-width broadening^96^. For aliphatic amino acids, CSA tensors tend to be smaller; hence we can expect a lower CSA impact^96,97^. However, most aliphatic fluorinated amino acids are toxic to *E. coli* and cannot be efficiently incorporated into proteins using bacterial expression^98^. To investigate whether our CF approach could be implemented to incorporate strategic ^19^F probes in amyloid prion fibrils, we tested the incorporation of two tailored fluorinated amino acids into the HET-s fold. In a recent study, we reported a general fluoroalkylation method to chemically synthesize bis-trifluoromethylated compounds that was successfully applied to the stereoselective synthesis of 5,5,5,5’,5’,5’-hexafluoro-l-leucine (Hfl), an analog of Leucine in which the two methyl groups are replaced by cognate trifluoromethyl groups^99^. HET-s PFD has three leucines (L241, L250, and L276), which can potentially lead to six C^19^F_3_ signals, in case of no structural polymorphism. We employed CF synthesis to produce a fluorinated HET-s sample (called here CF-6F-HETs) incorporating Hfl as a substitute for leucine. We applied the same CF protein synthesis conditions as used for CF-GVI-HET-s. The CF approach is very cost-effective to introduce fluorinated amino acids compared to regular bacterial overexpression, because we used small amounts of fluorinated amino acids (~10 mg for our CF method) compared to ~100 mg for cell-based methods^98^. We obtained a yield of ~1 mg of protein per mL of RM.

**Fig. 3:**
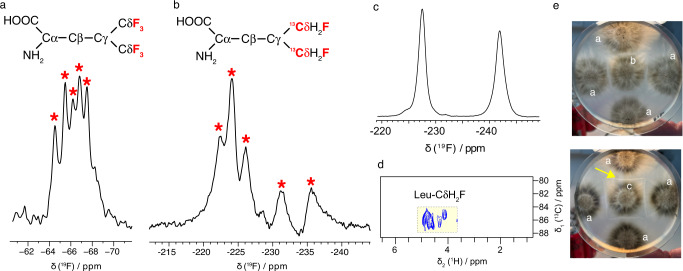
Solid-state NMR analysis and prion infectivity of fluorinated amyloid fibrils. ^19^F NMR at 60 kHz MAS experiments of cell-free synthesized HET-s labeled with (**a**) 5,5,5,5’,5’,5’-hexa-fluoro-Leucine (CF-6F-HETs) and **b** 5,5’-difluoro-L-leucine-5,5’-^13^C (CF-2F-13C-HETs). **c** ^19^F MAS NMR experiment recorded on 5,5’-difluoro-l-leucine-5,5’-^13^C. **d** 2D hCH experiment recorded on CF synthesized CF-2F-C-HETs. **e** Transfection of CF-synthesized and fluorinated HET-s aggregates induces conversion of [Het-s*] strains to the [Het-s] prion-infected state. The central strain is the focal strain and is confronted four times with a [Het-S] tester (marked a). The upper plate is an example of a no protein control. The focal strain (marked b) retained the prion-free [Het-s*] state and leads to a diffuse contact line with the [Het-S] tester (no barrage). The lower plate is an example of a strain transfected with CF-synthesized fluorinated HET-s. The focal strain (marked c) acquired the prion-infected [Het-s] state and produces a sharp contact line with the [Het-S] testers (barrage reaction, marked with yellow arrow).

The ^19^F experiment recorded on CF-6F-HETs (Fig. 3a) at 60 kHz MAS using a 1.3 mm rotor shows a peak pattern with resonances spreading over ~5 ppm. The pattern is well-resolved, especially considering the broadening usually observed for protein samples using ^19^F detection. Indeed, the strong dipolar couplings between ^19^F nuclei and between ^19^F and ^1^H nuclei usually compromise the spectral resolution and thus the application of ^19^F detection to biomolecular samples. We observed narrow ^19^F lines (~100–160 Hz), indicative of a relatively well-ordered conformation of these three fluorinated leucines in the HET-s assembly. Previously the measured ^19^F line-widths for fluorine labeled aromatic amino acids in proteins were ~140–600 Hz^100^. Here, we demonstrate that the use of MAS at fast regime (60 kHz) combined with a tailored ^19^F spin dilution (six ^19^F sites introduced by the CF synthesis) leads to a 1D experiment where five out of six C^19^F_3_ signals for the three residues can be resolved.

Next, we implemented the simultaneous incorporation of ^13^C and ^19^F nuclei at the level of the same amino acid 5,5’-difluoro-L-leucine-5,5’-^13^C^101^ (sample termed called CF-2F-C-HETs). This strategic labeling on leucine has two main advantages: (i) we re-introduce two ^1^H spins on the methyl sites, to enable efficient ^1^H-^13^C polarization transfers to the methyl carbon, (ii) while we keep two fluoromethylated spins for ^19^F detection and (iii) we introduce a ^13^C isotope directly attached to ^19^F spins to shift ^13^C signals away from the protonated methyl region and allow recording of 2D ^13^C-^1^H or ^13^C-^19^F correlations for increased resolution. A 1D ^19^F experiment recorded on CF-2F-C-HETs (Fig. 3b) at 60 kHz MAS using a 1.3 mm rotor displays 5 distinct chemical shifts, which were in a wider range compared to what is observed in a spectrum of pure 5,5’-difluoro-L-leucine-5,5’-^13^C (Fig. 3c). Remarkably, the ^19^F chemical shift dispersion for CF-2F-C-HETs was ~20 ppm, much higher compared to protein samples with ^19^F labeled aromatic amino acids^100^. We observed the CH_2_^19^F signals in a range of −220 to −240 ppm, in agreement with solution ^19^F NMR study by Otting et al.^101^, this range being very different compared to the C^19^F_3_ range (−65 to −70 ppm). We observed ^19^F line widths of ~1000 Hz. For the CF-2F-C-HETs sample, the ^19^F line widths are broader due to close ^1^H nuclei on the methyl site, associated with strong dipole couplings, which contribute to the homogenous component of the ^19^F line width. The ^19^F line width can be substantially improved by even faster MAS rates in combination with ^1^H decoupling^102^.

As observed for the CF-6F-HETs sample, the experimental ^19^F line width and the number of observed resonances suggest that the sample is highly homogeneous. Overall, the 1D spectral quality in the ^19^F dimension of our CF synthesized samples is excellent and in the range of reported MAS NMR data obtained on small compounds^102–105^, amino acids^106^, peptides^107,108^ and protein samples^100,109–112^ from the Polenova, Gronenborn and Hong laboratories at various magnetic fields and MAS frequencies. Next, to investigate the local effect of fluorine incorporation into the ^13^C signals, we conducted a 2D hCH experiment on CF-2F-C-HETs. We observed a strong chemical shift perturbation based on fluorinated leucine ^13^C signals detected at ~84–88 ppm (Fig. 3d), while Cδ chemical shifts are usually observed in the range of ~24–26 ppm in protonated protein samples. ^1^H leucine signals in CF-2F-C-HETs are also shifted from ~0.73 ppm in protonated samples to a range of ~4-5 ppm. In total 6 signals can be distinguished in the 2D spectrum. The observation of distinct fluorinated methyl ^13^C signals together with the chemical shift ^13^C and ^1^H dispersion as measured in the CF-2F-C-HETs sample offer advantageous spectroscopic features. As demonstrated here for the HET-s fibrillar assembly, the presented CF approach is useful to incorporate fluorinated ^13^C-labeled methyl residues for a spectral identification. To validate that the fluorinated version of HET-s PFD is also acting as a functional prion in *Podospora anserina*, we conducted a prion infectivity test (Fig. 3e), and we observed the same infectious properties as fibrils assembled in vitro from purified HET-s. These results indicate that the incorporation of fluorine atoms in the HET-s fold is not affecting its biological functions in *Podospora anserina*.

## Conclusion

In this study, we report the cell-free synthesis of amyloid fibrils for structural investigations. We demonstrate that yield of ~1 mg of protein per mL of CF reaction mixture could be obtained using a straightforward protocol in a cost-effective way. The total sample preparation time was around 19 h. Interestingly, we observed that monomers of HET-s directly self-assembled in the reaction mixture to form amyloid fibrils. NMR experiments reveal the absence of structural polymorphism in the amyloid fold of CF synthesized HET-s, thus the approach can substantially reduce the complexity of amyloid protein purification and in vitro assembly. The CF-synthesized fibrillar assemblies exhibit the structural and biological features of the infectious amyloid form of HET-s amyloids obtained in vitro from purified samples. A similar observation has been made by Weliky and coworkers on influenza virus FHA2 protein purified from inclusion bodies^113^.

**Fig. 4:**
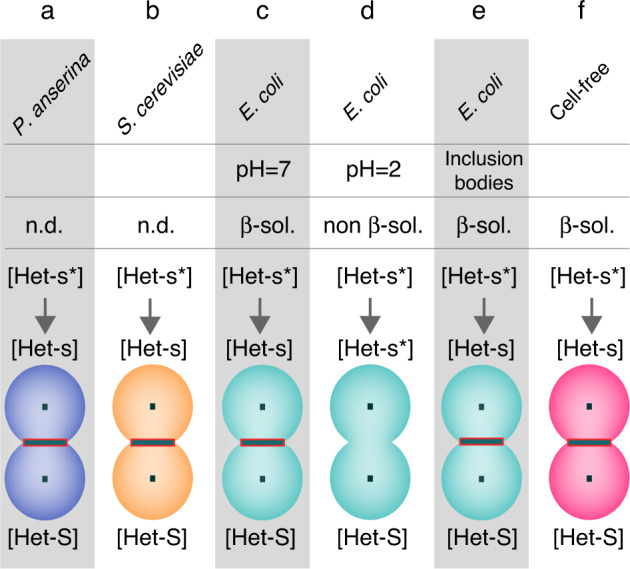
Infectivity of the prion-forming domain of HET-s from various cell culture and assembly conditions. Summary of the infectivity of HET-s (218-289) aggregates depending on the culture and assembly conditions. **a**, **b** HET-s expressed in *P. anserina* and *S. cerevisiae* from refs. 80, 114. **c**, **d** HET-s aggregates purified from an *E. coli* expression and assembled at pH = 7^84^ and pH = 2^11,71^. **e** HET-s aggregates extracted from *E. coli* inclusion bodies from ref. 79. **f** HET-s assembled in the CF reaction mixture from this work. High-resolution structures for (**a** and **b**) are unknown. **c**, **e**, **f** aggregates present a β-solenoid fibrillar structure. **d** aggregates are not β-solenoid. **a**–**c**, **e**, **f** aggregates are infectious, they induce the conversion of [Het-s*] non-prion strains to prion-infected in [Het-s] strains that produce a barrage reaction to [Het-S]. **d** aggregates are not infectious.

Figure 4 summarizes the assembly-function relationship for the reference amyloid prion HET-s. The “in cell” conformation of HET-s aggregates in the native host *P. anserina* and in *S. cerevisiae* are still unknown, both induce the conversion of non-prion strains to the [HET-s] prion state. In this work, we demonstrate that CF-synthesized aggregates have the structural features of a β-solenoid amyloid fold, comparable to HET-s (218–289) PFD aggregates purified from *E. coli*, including aggregates obtained from the biochemical extraction of inclusion bodies. The biological features of CF-synthesized aggregates also correspond to the infectious form of HET-s aggregates that can be induced in vivo in *P. anserina*, as observed by the conversion of prion-free to prion-carrying strains ([Het-s*] state to towards [Het-s] state) induced by CF-synthetized aggregates. This biological ability is a hallmark of the functional amyloid HET-s and similar observations have been made for aggregates extracted from bacterial inclusion bodies and yeast heterologous expression extracts. The CF expression condition differs both from the simple, low molecular crowding-controlled conditions of in vitro assembly from purified protein and from the native or heterologous in cellulo conditions. Nevertheless, the CF-expressed protein assembled into highly ordered, structurally homogenous and biologically relevant fibrils. HET-s is known to aggregate into stable and well-defined amyloid structure at the local level, and thus offered us a favorable model to implement the cell-free synthesis of functional amyloids. Further work will be required to investigate if the low structural polymorphism observed for CF-synthesized HET-s is a general feature, especially in the context of pathological amyloids that are rather prone to adopt polymorphic structures.

The synthesis yield of ~1 mg is amenable to modern solid-state NMR analysis by taking advantage of fast MAS regimes >60 kHz. We demonstrated that high-resolution 2D fingerprints employing ^13^C and ^1^H dimensions could be obtained on CF amyloid fibrils. These structural fingerprints nowadays offer powerful tools to rapidly investigate the conformation of fibrillar protein assemblies, as we recently demonstrated for pathological and functional amyloids^25,39^. We showed that 3D spectroscopy could be applied to CF-synthesized amyloid fibrils as well. Ultimately, we demonstrated the production of fluorinated amyloid fibrils using the CF approach, amenable to ^19^F solid-state NMR for further structural studies. The incorporation of fluorinated side chains in the HET-s amyloid fold may have led to significant changes in the structural architecture. Indeed, the incorporation of fluorinated methyl residues increases the hydrophobicity, the dipole moment, and the steric volume compared to native hydrogenated residues. The incorporation of 5,5,5,5’,5’,5’-hexa-fluoro-L-leucine and 5,5’-difluoro-L-leucine-5,5’-^13^C into the cell-free synthesis protocol do not change the expression yield, and we could retrieve self-assembled fluorinated HET-s samples using a similar washing procedure. Moreover, our NMR results demonstrate that the non-polymorphic amyloid fold is conserved for fluorinated, CF-synthesized HET-s. Our approach demonstrated here on the model amyloid system HET-s, allows for the production of amyloid fibrils with tailored amino acid isotope labeling and will open an avenue to further characterize complex fibrillar protein assemblies.

## Methods

### Plasmids and strains

The DNA templates of HET-s (218-289) were ligated into the pIVEX2.3 expression vector using *Xho*I and *Nde*I cloning sites and ligated using T4 DNA ligase. For in cell recombinant expression in *E. coli*, the HET-s(218-289) DNA fragment was inserted into the pET21 vector. The plasmids were sequenced by the Eurofins company. For the large-scale preparation of CF synthesis, the plasmid of HET-s (218-289) was cloned in the DH5alpha bacterial strain and prepared using the MAXI prep kit from Macherey-Nagel.

### Cell-free protein synthesis

The *E. Coli* CFS S30 extract was prepared as described before^74^. The HET-s GVI ^13^C,^15^N-labeled sample was prepared using a membrane dialysis protocol according to ref. ^74^. The master mix, feeding buffer and reaction mixtures were prepared for one reaction with added sample amounts summarized in Supplementary Tables S1–S3. The feeding mix was prepared by mixing the master mix with S30 buffer (10 mM Tris acetate pH 8.2, 14 mM Mg(OAc)_2_ 0.6 mM KOAc and 1 mM DTT) and AA-mixture and was pre-incubated at 30 °C. Note that although not optimized in our study, the plasmid design, the concentration of salts, the codon usage, temperature, the viscosity and the ATP regenerating system are among the most critical conditions for a good expression. The reaction mixture (1 mL for the GVI sample in a 0.7 mm rotor, 3 mL for the CF-6F-HET-s and CF-2F-13C-HETs samples in 1.3 mm rotors) was transferred to a 6–8 kDa dialysis membrane, then transferred into a 50 mL falcon tube containing 17 mL (for the GVI sample and 51 mL for the CF-6F-HET-s and CF-2F-13C-HETs samples) of pre-warmed (at 30 ^o^C) feeding buffer. The mixture was dialyzed for 18 h at 30 °C, 120 rpm shaking. After the dialysis, the reaction mixture was harvested from the dialysis membrane. The mixture was centrifuged 30 min, 12,000 rpm at room temperature. The supernatant was removed and the pellet was washed three times with 1 mL of distilled H_2_O by centrifuging each step for 10 min, 12,000 rpm at room temperature. The amino-acid mixture was prepared by weighting each amino acid separately. All amino acids were unlabeled except for G, V, and I, which were uniformly (^13^C,^15^N)-labeled.

### Synthesis of fluorinated compounds

Enantiopure 5,5,5,5’,5’,5’-hexafluoro-L-leucine (e.r. >99:1) was synthesized using a recently reported methodology featuring a direct stereoselective incorporation of the hexafluoroisobutyl group on a chiral glycine Shiff base nickel complex using the 2-(bromomethyl)−1,1,1,3,3,3-hexafluoropropane reagent^99^.

5,5’-difluoro-L-leucine-5,5’-^13^C was synthesized with the methodology described in ref. ^101^.

### In vivo recombinant HET-s production

The recombinant pET21-HET-s(218-289) plasmid was transformed in the *E.coli* BL21(DE3) pLysS bacteria. The expression in M9 minimal media was carried out at 37 °C and induced with IPTG at OD_600_ = 0.8 and the expression was continued for 4 h. The cells were harvested by centrifugation, then lyzed and incubated overnight at 60 °C, sonicated and ultra-centrifuged. The supernatant was purified in a 5 mL Histrap HP column (GE Healthcare). The sample was eluted with 50 mM Tris, 0.5 M NaCl, 500 mM imidazole, 7 M urea, pH 8. The HET-s protein was buffer exchanged into 150 mM acetic acid pH 3.0, using a HiPrep 26/10 desalting column (GE Healthcare). Aggregation was performed by adding (12% v/v) 3 M Tris-Cl pH = 8 buffer in 150 mM acetic acid until pH = 7, then left for agitation at room temperature for several days.

### SDS-PAGE

All SDS-PAGE gels were made from 13% acrylamide and stained in the 5% Coomassie blue solution. The samples from pellets were first re-solubilized in the 8 M urea solution.

### Negative staining electron microscopy

Samples were stained with a 2% uranyl acetate (w/v) solution for 1 min and dried under dark condition. Samples were observed in an FEI CM120 transmission electron microscope at an accelerating voltage of 120 kV under TEM low-dose mode. TEM images were recorded using a Gatan USC1000 2 k 9 2 k camera (Pleasanton, CA, USA).

### Het-s infectivity

Het-s protein transfection experiments were performed as described previously using a cell disruptor (Fast-prep FP120, Bio101, Qbiogen Inc.)^71^. For each test, ~0.5 cm^3^ of [Het-s*] mycelium grown on solid medium is sheared (run time 20 s, 6 m s^−1^) in 450 ml of STC50 buffer (0.8 M sorbitol, 50 mM CaCl_2_, 100 mM Tris HCl pH 7.5) and 50 μl of sample in a 2 ml screw-cap tube. The sheared mycelium is then plated onto D0 medium (40 μl by spot at a distance of 2 cm of [Het-S] strain) and incubated at 26 °C until direct confrontation with the [Het-S] strain occurred (~5 days), and the number of strains producing a barrage reaction to [Het-S] was counted.

### NMR rotor sample packing

CF-synthetized amyloid fibrils were packed into a 1.3 mm or 0.7 mm rotor, using Giottobiotech filling tools, after a step of ultracentrifugation for 18 h, 4 °C at 25,000 RPM with a swinging bucket rotor (32SWTi, Beckman ultracentrifuge).

### Solid-state NMR spectroscopy

Solid-state NMR experiments were performed on 18.8 T (for ^1^H detection), and 11.7 T Bruker spectrometers (for ^19^F detection), and the BCU temperature was set to 269 K, resulting in a sample temperature of ~293 K. All NMR data acquisition and processing parameters are summarized in Supplementary Tables S6 and S7. For all 2D and 3D experiments, the MISSISSIPPI water suppression scheme was applied. The ^19^F experiments were conducted at 60 kHz MAS using a 1.3 mm HX probe in which ^1^H channel was configured for ^19^F detection. The ^19^F spectra for protein samples were acquired with an interscan delay of 1.5 s, ns = 32 k, aq = 8.7 ms, resulting in a total acquisition time = 17 h. Trifluoroacetic acid was used for ^19^F chemical shift referencing. The ^19^F line widths were measured using deconvoluted lines from spectra. No apodization functions were applied for extracting ^19^F line widths. All spectra were processed with Bruker TopSpin 3.6 and analyzed with the CcpNmr 2.4.2 software.

### Statistics and reproducibility

Cell-free synthesis of HET-s was performed for unlabeled, (^19^F-labeled), (^13^C, ^19^F-labeled), and (^13^C, ^15^N-labeled) samples. Attempts for replication of the cell-free synthesis protocol were successful. Multi-dimensional solid-state NMR experiments were recorded in building blocks (2–8 times) and added together.

### Reporting summary

Further information on research design is available in the Nature Portfolio Reporting Summary linked to this article.

## Supplementary information

Supplementary Information

Reporting Summary

## Data Availability

The data that support the findings of this study are available from A. Lo upon request. The precise medium composition for the master mixture, reaction mixture, and feeding mixture are given in the Supporting Information.
