# Supplementary material for: Cell-free synthesis of amyloid fibrils with infectious properties and amenable to sub-milligram magic-angle spinning NMR analysis
Source: Commun Biol. 2022 Nov 9;5:1202. doi: 10.1038/s42003-022-04175-1 (PMC9646696; doi:10.1038/s42003-022-04175-1)
Supplement: Supplementary file 1 — Supplementary Information [file 42003_2022_4175_MOESM1_ESM.pdf]

Cell-free synthesis of amyloid fibrils with infectious properties and amenable to sub-milligram magic-angle spinning NMR analysis

Alons Lends[1]*, Asen Daskalov[1,2,5], Ansis Maleckis[3], Aline Delamare[1], Mélanie Berbon[1], Axelle Grélard[1], Estelle Morvan[4], Jayakrishna Shenoy K.P.[1], Antoine Dutour[1], James Tolchard[1], Abdelmajid Noubhani[1], Marie-France Giraud[1], Corinne Sanchez[1], Birgit Habenstein[1], Gilles Guichard[1], Guillaume Compain[1], Kristaps Jaudzems[3], Sven J. Saupe[2], Antoine Loquet[1]*

[1] Univ. Bordeaux, CNRS, Bordeaux INP, CBMN, UMR 5248, IECB, Pessac, France
[2] Univ. Bordeaux, CNRS, IBGC, UMR 5095, Bordeaux, France
[3] Latvian Institute of Organic Synthesis, Riga, Latvia
[4] Univ. Bordeaux, CNRS, INSERM, IECB, UAR 3033, Pessac, France
[5] Present address: Zhejiang Academy of Agricultural Sciences, Hangzhou, 310021, China

Supplementary information

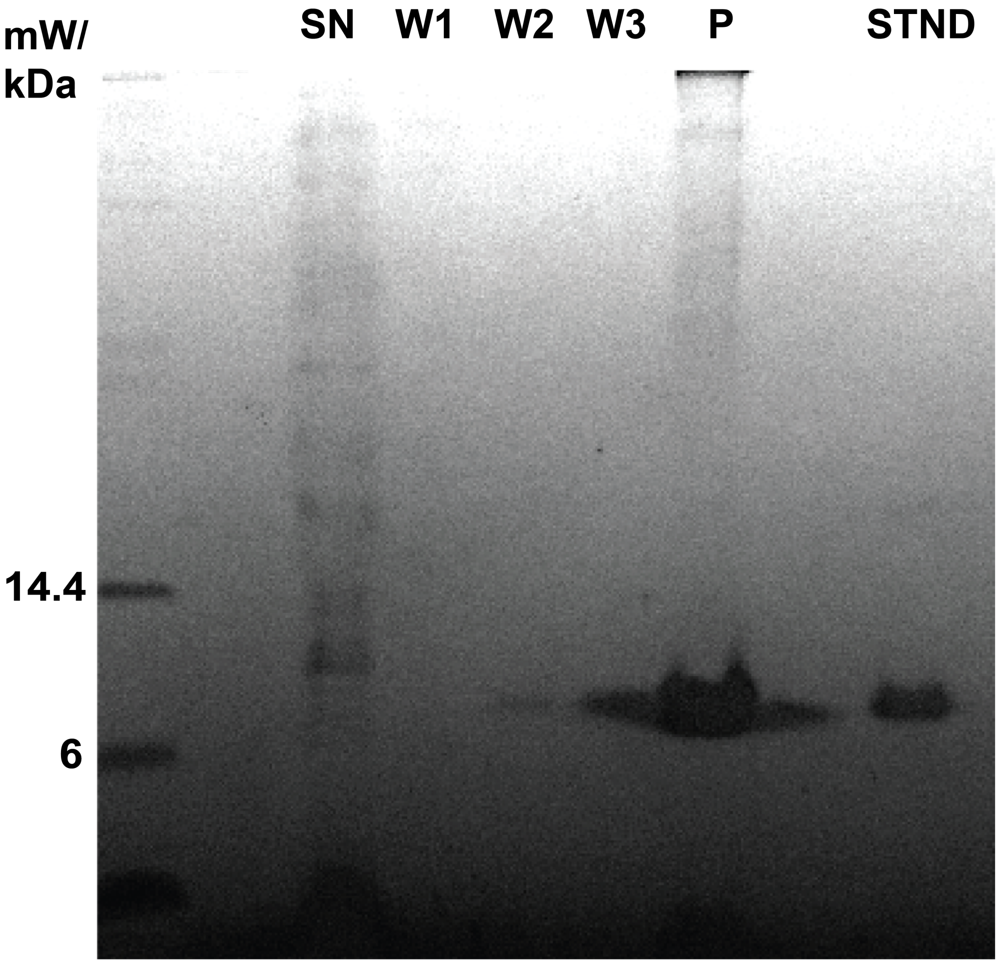

Figure S1: SDS-PAGE gel of cell-free synthesized HET-s (218-289). SN: supernatant. W1, W2, W3: washing fractions 1, 2 and 3, P – washed pellet. Standard (Stnd) is the recombinant HET-s (218-289) protein, with a molecular weight of 9.8 kDa. The pure HET-s (218-289) protein was found in the pelleted fraction.

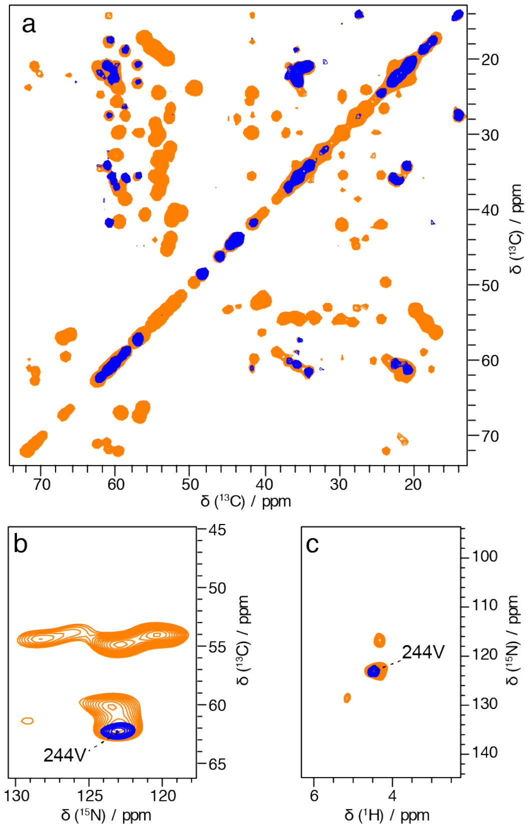

Figure S2: (a) Overlay of 2D ^{13}C-^{13}C projections from the 3D hCCH TOCSY of R-HET-s PFD (in orange) and CF-GVI-HET-s (in blue). (b) 2D ^{13}C-^{15}N and (c) ^{15}N-^{1}H of the 3D hNCaH of R-HET-s PFD (in orange) and CF-GVI-HET-s (in blue). Intra-residue correlations for V244 are highlighted.

Table S1. Reagents and concentrations for the master mixture (MM).

Reagent	Stock	Final	V, μL
NaN_3	10 % w/v	0,05%	90
PEG 8000	40 % w/v	2%	900
KOAc	4 M	150.8 mM	679
$Mg(OAc)_2$	1 M	7.1 mM	332
HEPES	2.5 M	0.1 M	660
Complete*	50 x	1 x	360
Folinic Acid	10 mg/ml	0.1 mg/ml	180
DTT	0.5 M	2 mM	72
NTP mix**	75 x	1 x	240
PEP	1 M	20 mM	360
Acetyl Phosphate	1 M	20 mM	360
RCWMDE	17 mM	1 mM	1078
AA-mix	5 mM	1 mM	2250
H_2O			1800
Total			9211

* Complete protease inhibitor mixture was prepared by dissolving 1 tablet in 1 mL H_2O.

**NTP mixture was prepared by mixing together 1.25 mL of 0.36 mM ATP, 1.25 mL of 0.24 mM CTP, 1.25 mL 0.24 mM GTP and 1.25 mL of 0.24 mM UTP.

Table S2. Reagents and concentrations for the feeding (FM).

Reagent	Stock	Final	V, μL
MM			8699
S30 Buffer	100%	30%	5950
AA-mix	5 mM	0.5 mM	2125
H_2O			226
Total			17000

Table S3. Reagents and concentrations for the reaction mixture (RM).

Reagent	Stock	Final	V, μL
MM			512
Pyruvate kinase	10 mg/ml	0.04 mg/ml	8
tRNA	40 mg/ml	1 mg/ml	12
T7 RNAP	420 U/µl	6 U/µl	14.3
RNA Guard	32 U/µl	0.3 U/µl	10.7
DNA	1000 µg/ µl	50 ng/µL	50
S30 extract	100%	30%	350
H_2O			47
Total			1000

Table S4. The extracted line widths (at FWHM) for ^{1}H amide resonances in both samples.

Residue	CFS	R-HET-s PFD
	^{1}HN Line widths/Hz	^{1}HN Line widths/Hz
244V	250	390
245V	252	390
264V	254	499
267V	252	366
268V	315	585
271I	267	315
275V	485	390
277I	218	476
278G	226	275
282G	301	377
283G	238	311
285G	163	355

**Average**	**268±78**	**394±87**

Table S5. The extracted line widths (at FWHM) for ^{1}Ha resonances in both samples.

Residue	CFS	R-HET-s PFD
	^{1}Ha Line widths/Hz	^{1}Ha Line widths/Hz
**231I**	155	195
**239V**	204	259
**244V**	269	269
**245V**	417	227
**264V**	141	228
**267V**	201	239
**268V**	251	259
**277I**	161	190
**Average**	225±90	223±29

Table S6. The experimental NMR parameters for 2D spectra.

	CFS GVI		R-HET-s PFD	
**Experiment**	**2D hNH**	**2D hCH**	**2D hNH**	**2D hCH**
**MAS frequency/kHz**	100	100	100	100
**Field/T**	18.8	18.8	18.8	18.8
**Transfer I**	HN-CP	HC-CP	HN-CP	HC-CP
**^{1}H field/kHz**	80	80	80	80
**^{15}N field/kHz**	20		20	
**^{13}C field/kHz**		20		20
**Shape**	ramp ^{1}H	ramp ^{1}H	ramp ^{1}H	ramp ^{1}H
**Time/ms**	1.3	0.3	0.9	0.3
**Transfer II**	NH-CP	CH-CP	NH-CP	CH-CP
**^{1}H field/kHz**	80	80	80	80
**^{15}N field/kHz**	20		20	
**^{13}C field/kHz**		20		20
**Shape**	ramp ^{1}H	ramp ^{1}H	ramp ^{1}H	ramp ^{1}H
**Time/ms**	0.7	0.3	0.8	0.3
**^{13}C carrier/ppm**		54		54
**^{1}H decoupling**	slTPPM12	slTPPM12	slTPPM12	slTPPM12
**^{1}H decoupling field/ kHz**	25	25	25	25
**t1 increments**	128	300	74	400
**Windows function**	QSine 3	QSine 3	QSine 3	QSine 3
**Sweep width (t1)/kHz**	32.4	18.1	42.4	18.1
**Acquisition time (t1)/ms**	19.7	11	11.4	11
**^{15}N/^{13}C decoupling**	WALTZ16	WALTZ16	WALTZ16	WALTZ16
**^{15}N/^{13}C decoupling field/ kHz**	10		10	10
**t2 increments**	1536	1536	1536	1536
**Windows function**	QSine 3	QSine 3	QSine 3	QSine 3
**Sweep width (t2)/kHz**	100	37	100	37
**Acquisition time (t2)/ms**	10.2	20.7	10.2	20.7
**Interscan delay/s**	1.3	1.6	1.3	1.6
**Number of scans**	64	32	32	32
**Measurement time/h**	1.5	5.7	0.75	5.7

Table S7. The experimental NMR parameters for 3D spectra

	CFS GVI		R-HET-s PFD	
**Experiment**	**3D hNCaH**	**3D hCCH**	**3D hNCaH**	**3D hCCH**
**MAS frequency/kHz**	100	100	100	100
**Field/T**	18.8	18.8	18.8	18.8
**Transfer I**	NH-CP	HC-CP	NH-CP	HC-CP
**^{1}H field/kHz**	80	80	80	80
**^{13}C field/kHz**		20		20
**^{15}N field/kHz**	20		20	
**Shape**	ramp ^{1}H	ramp ^{1}H	ramp ^{1}H	ramp ^{1}H
**Time/ms**	1.8	0.4	1.4	0.4
**Transfer II**	NC-CP	TOCSY	NC-CP	TOCSY
**^{15}N field/kHz**	20		20	
**^{13}C field/kHz**	80	25	80	25
**Shape**	ramp ^{15}N		ramp ^{15}N	
**Time/ms**	11	14	11	14
**^{13}C carrier/ppm**	54	40	54	40
**^{1}H decoupling**	slTPPM12			slTPPM12
**^{1}H decoupling field/ kHz**	25	25		25
**Transfer III**	CH-CP	CH-CP	CH-CP	CH-CP
**^{1}H field/kHz**	80	80	80	80
**^{13}C field/kHz**	20	20	20	20
**Shape**	ramp ^{1}H	ramp ^{1}H	ramp ^{1}H	ramp ^{1}H
**Time/ms**	0.4	0.3	0.3	0.3
**t1 increments**	32	102	40	124
**Windows function**	QSine2	QSine 3	QSine 3	QSine 2
**Sweep width (t1)/kHz**	10.4	12.4	8	14.1
**Acquisition time (t1)/ms**	3.3	4.1	2.5	4.4
**^{1}H decoupling**	slTPPM12	slTPPM12	slTPPM12	slTPPM12
**^{1}H decoupling field/ kHz**	25	25	25	25
**t2 increments**	48	104	40	124
**Windows function**	QSine 2	QSine 3	QSine 3	QSine 2
**Sweep width (t2)/kHz**	3.1	12.4	3.2	14.1
**Acquisition time (t2)/ms**	7.4	4.1	6.1	4.4
**^{15}N/^{13}C decoupling**	WALTZ16	WALTZ16	WALTZ16	WALTZ16
**^{15}N/^{13}C decoupling field/ kHz**	10	10	10	10
**t3 increments**	2048	2048	2048	2048
**Windows function**	QSine 3	QSine 3	QSine 3	QSine 3
**Sweep width (t3)/kHz**	81.9	48.5	82	48.5
**Acquisition time (t3)/ms**	12.5	21.1	12.5	21.1
**Interscan delay/s**	1.5	1.5	1.5	1.2
**Number of scans**	64	4	24	4
**Measurement time/h**	41	17.7	16	20.5
